# Eicosapentaenoic Acid and Medium-Chain Triacylglycerol Structured Lipids Improve Endurance Performance

**DOI:** 10.3390/nu15173692

**Published:** 2023-08-23

**Authors:** Katsunori Tsuji, Yosuke Tsuchiya, Kaori Yokoi, Kenichi Yanagimoto, Hisashi Ueda, Eisuke Ochi

**Affiliations:** 1 Sports Research Center, Hosei University, Kawasaki 211-0065, Japan; katsunori.tsuji.78@hosei.ac.jp; 2 Center for Liberal Arts, Laboratory of Health and Sports Sciences, Meiji Gakuin University, Yokohama 244-8539, Japan; yosuket@gen.meijigakuin.ac.jp; 3 Food Function R&D Center, Nissui Corporation, Tokyo 105-8676, Japan; kaori_yokoi@nissui.co.jp (K.Y.); yanagimoto@nissui.co.jp (K.Y.); 4 Faculty of Health and Medical Science, Department of Medical Sports, Teikyo Heisei University, Ichihara 290-0193, Japan; h.ueda@thu.ac.jp; 5 Faculty of Bioscience and Applied Chemistry, Hosei University, Tokyo 184-8584, Japan; 6 Graduate School of Sports and Health Studies, Hosei University, Tokyo 194-0298, Japan

**Keywords:** eicosapentaenoic acid, medium-chain triacylglycerols, structured lipids, endurance, performance

## Abstract

Purpose: The effects of intake of STGs containing esterified eicosapentaenoic acid (EPA) and medium-chain triglycerides (MCTs) on cardiorespiratory endurance have not yet been reported. This study aimed to examine the efficacy of interesterified structured lipids EPA and MCTs on cardiorespiratory endurance. Methods: This 8-week randomized double-blind placebo-controlled parallel-group study involved 19 healthy men. The participants were randomly assigned to a group that received interesterified structured lipids EPA and MCTs (STG group, 9 participants) or a group receiving a PM of EPA and MCTs (PM group, 10 participants). The outcome measures were time to exhaustion (TTE) and time to reach the anaerobic threshold in the peak oxygen uptake (VO_2_peak) test, VO_2_peak, and anaerobic threshold. Results: The increase in TTE in the VO_2_peak test after the intervention period compared with before the intervention period was significantly greater in the STG group (53 ± 53 s) than in the PM group (−10 ± 63 s; *p* < 0.05). Similarly, the increase in time to reach the anaerobic threshold was significantly greater in the STG group (82 ± 55 s) than in the PM group (−26 ± 52 s; *p* < 0.001). Conclusion: This study demonstrated that the consumption of interesterified structured lipids EPA and MCTs improved endurance in humans.

## 1. Introduction

Omega-3 fatty acids such as eicosapentaenoic acid (EPA) and docosahexaenoic acid (DHA) are widely known to be effective in improving cardiovascular function, lowering blood pressure, and improving cognitive function [1,2,3,4,5,6]. In addition, EPA is reported to improve erythrocyte deformability [7,8,9], facilitate fatty acid beta-oxidation (FAO) [10,11], and decrease both oxygen consumption and heart rate during exercise, which suggests it may have endurance-enhancing effects [12]. Continuous intake of EPA is expected to improve blood circulation, increase oxygen delivery capacity, and enhance the oxygen utilization rate, thereby raising the body’s maximal oxygen uptake [13]. However, no consensus has been reached on the effect of EPA and DHA intake on maximal oxygen uptake. A recent study reported that 8 weeks of EPA (140 mg) and DHA (560 mg) intake improved the economy of cycling during a physiologically demanding time trial [14]. In addition, a study of male elite cyclists reported significant increases in maximal oxygen uptake after a daily intake of 0.66 g EPA and 0.44 g DHA for 3 weeks [13]. However, a study of male soccer players did not report any differences in maximal oxygen uptake after a daily dose of 1.6 g EPA and 1.0 g DHA for 10 weeks [15]. A recent narrative review stated that previous studies on the effects of EPA/DHA intake on endurance performance are few and inconsistent [16].

The effects of the intake of medium-chain triglycerides (MCTs) on endurance have also been investigated. MCTs are tri-esters consisting of three medium-chain fatty acids and a glycerol molecule. MCTs are known to be more easily hydrolyzed and much more rapidly used for energy compared with long-chain triglycerides (LCTs) [17,18]. Thus, MCTs are reported to be effective in reducing body weight and body fat [19]. In addition, continuous intake of MCTs is reported to increase FAO and time to exhaustion (TTE) during endurance exercise, which suggests that MCTs may have endurance-enhancing effects [20,21]. Structured lipids are lipids in which specific fatty acids are bound to given sites in glycerol, which enhances the digestion and absorption characteristics and the physiological function of fats and oils. Structured lipids containing esterified MCTs and LCT such as linoleic acid have been studied to investigate their digestion and absorption in humans, their physiological properties, and applications against various diseases [22,23]. However, differences in effectiveness between the interesterified structured lipids EPA and MCTs (i.e., structured triglycerides [STGs]) and a physical mixture (PM) of EPA and MCTs have not yet been examined.

Effects on endurance are often examined using endurance tests, multi-stage incremental exercise tests, and the time to reach the anaerobic threshold (AT). A meta-analysis reported that carbohydrate supplementation before or during exercise increased carbohydrate storage in the body during exercise, thereby enhancing endurance performance [24]. Meanwhile, increased FAO during exercise resulting from lipid intake is likely to contribute to enhanced endurance by reducing carbohydrate oxidation during exercise. Indeed, Nosaka et al. reported that supplementation of MCT increases FAO during low-intensity exercise in a multi-stage incremental exercise test [25]. On the other hand, a recent systematic review indicates that most previous studies reported that MCT oil did not improve endurance performance and had no effect on respiratory exchange ratio, glucose concentration, fat and carbohydrate oxidation, and lactate concentration [16]. Therefore, it is possible that MCT may be more effective when consumed combined with fish oil rather than MCT alone. However, the effects of intake of STGs containing esterified EPA and MCTs on cardiorespiratory endurance have not yet been reported.

Thus, in this study, we hypothesized that the intake of STGs would improve the efficiency of lipid utilization during exercise, which would be accompanied by increases in TTE and peak oxygen uptake (VO_2_peak). The objective of this study was to test the efficacy of structured lipids containing esterified EPA and MCTs on cardiorespiratory endurance in sedentary male participants, using 2.1 g of a PM of EPA and MCTs as a placebo.

## 2. Materials and Methods

### 2.1. Participants

The participants were 19 untrained healthy men (Table 1). No obese individuals or females were included in this study. Females were not included in the participants due to menstrual cycle considerations. The participants were not allergic to fish and had not participated in any regular resistance training experience for at least one year before this study. Furthermore, the participants were asked not to participate in other clinical trials and interventions, including massages, stretching, strenuous exercise, excessive consumption of food or alcohol, and intake of supplements or medication, during the experimental period. All the participants were provided with detailed explanations of the study protocol prior to participation, and informed consent was obtained from all the participants. The study was conducted in accordance with the Declaration of Helsinki and was approved by the ethics committee for human experiments at Teikyo Heisei University (ID: 2021-01358276). The study was registered at the University Hospital Medical Information Network Clinical Trials Registry (UMIN-CTR identifier: UMIN000044958).

### 2.2. Study Design

**Figure 1:**
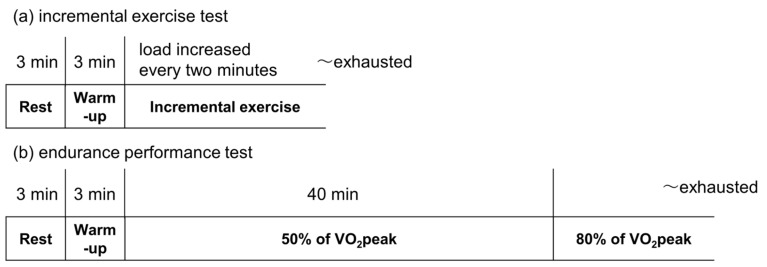
Protocols for the exercise tests. (**a**) Incremental exercise test. (**b**) Endurance test.

The study used a double-blind, placebo-controlled, parallel-group trial design. The participants were randomly assigned to two groups using a table of random numbers to minimize intergroup differences in terms of age, body mass index (BMI), and VO_2_peak. The participants consumed either STGs containing esterified EPA and MCTs or a PM of EPA and MCTs daily for 8 weeks before and after the exercise experiment. Sequence allocation concealment and blinding of participants and researchers were maintained throughout this period. Medication adherence was assessed using the daily records of the participants and via pill counts performed at the end of the study. The exercise test was performed over 2 days. On the first day, VO_2_peak was measured. An endurance test was performed at least 48 h after the VO_2_peak measurement (Figure 1). The participants were instructed to eat a light meal > 2 h before arriving at the laboratory. In addition, they were asked to refrain from any exercise for 24 h before the study visit. The primary outcomes were the TTE in the VO_2_peak test and the endurance test. Secondary outcomes included VO_2_peak, AT, TTE at AT in the VO_2_peak test, TTE in the endurance test at a workload of 80% of VO_2_peak, and total workload in the endurance test. We also examined the nutrition status of all the participants before and after the supplement intake, using the Food Frequency Questionnaire Based on Food Groups Ver. 6 (FFQg; Kenpakusha, Tokyo, Japan). This experiment was conducted in a laboratory in Tokyo, Japan.

### 2.3. Supplements

The STG group consumed interesterified structured lipids EPA and MCTs, while the PM group consumed EPA and MCTs mixed in oil. Both groups consumed 10 soft capsules (Nissui Corporation, Tokyo, Japan) per day for 8 weeks plus 2 days, and the total consumption was 4380 mg per day (600 mg EPA and 260 mg DHA). The subjects consumed the capsules with water.

### 2.4. Measurements of Peak Oxygen Uptake (VO_2_peak)

The participants visited the laboratory four times throughout the experimental period. During the first visit, subjective VO_2_peak was determined using an incremental exercise test performed on a cycle ergometer (Aerobike 75XLIII; Combi Wellness Corp., Tokyo, Japan) at 60 revolutions per min (rpm). The test began at 60 W and increased by 30 W every 2 min until exhaustion. When the participant’s pedal rotation speed dropped below 50 rpm three times, the test was ended. The duration of exercise was recorded at the end of the exercise test. The maximal value of VO_2_ observed during the exercise was defined as VO_2_peak. Respiratory gases were collected and analyzed using an automatic gas analyzer (AE-300S; Minato Medical Science Co., Ltd., Tokyo, Japan). The collected data were averaged every 30 s.

### 2.5. Measurements of Anaerobic Threshold (AT)

AT was determined from the point at which (i) the rate of increase in carbon dioxide emissions increased relative to the rate of increase in oxygen uptake with increasing exercise intensity, or (ii) the rate of increase in ventilation rapidly increased during the VO_2_peak test.

### 2.6. Measurements of Endurance Test

The endurance tests were conducted at least 2 days after the VO_2_peak measurement. The endurance test was performed using a cycle ergometer at 60 rpm. After 3 min of warm-up exercise at 20 W, the participants were allowed to exercise for 40 min at 50% of VO_2_peak. The intensity was increased to 80% of VO_2_peak, and the exercise was continued until exhaustion. The test was ended when the participant’s pedal speed dropped below 50 rpm three times. The duration of exercise was recorded at the end of the exercise.

### 2.7. Statistical Analyses

The values were expressed as means ± standard error. We hypothesized that TTE would change by 10 ± 10 min in the intervention group and 0 ± 5 min in the control group, based on the results of previous studies [21,26,27]. The effect size (ES) was calculated to be 1.265. Based on the above hypothesis, by estimating the number of participants required for α = 0.05 and a power of 80% in the analysis, 8 individuals per group were necessary for a total of 16 in both groups. Differences between the groups were compared using an independent two-sample *t*-test to evaluate the degree of change in all outcomes from baseline to week 8. A *p*-value of <0.05 was considered statistically significant. Analyses were performed using Sigma Plot 12 (Systat, San Jose, CA, USA). Data were collected from 8 August 2021 through 13 October 2021. The data analysis, which followed the intention-to-treat approach, was performed from 20 October 2021 to 1 November 2021.

## 3. Results

At baseline, no significant differences were observed between the groups in terms of age, weight, height, and BMI (Table 1). Based on the results of the FFQg, no differences were observed in either group between before and after supplementation.

### Experimental Trial

There was no significant difference in VO_2_peak before and after the intervention period between the STG and PM groups. The difference in AT before and after the intervention period in the STG group was significantly higher than that in the PM group (*p* < 0.01). However, there were no significant differences between the STG and PM groups in TTE and total workload during the endurance test before and after the intervention period. Compared with the difference in total workload during exercise at 50% of VO_2_peak in the endurance test before and after the intervention period in the PM group, that in the STG group was significantly higher (*p* < 0.05). However, there was no significant difference between the STG and PM groups in total workload during exercise at 80% of VO_2_peak before and after the intervention period (Table 2).

**Figure 2:**
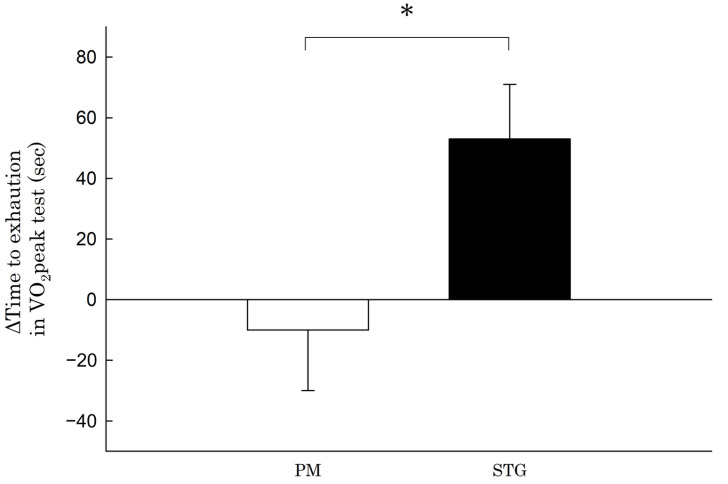
Change in the time to exhaustion in the VO_2_peak test. PM: physical mixture; STGs: structured triglycerides. * Statistically significant difference from the value in the STG group (*p* < 0.05).

**Figure 3:**
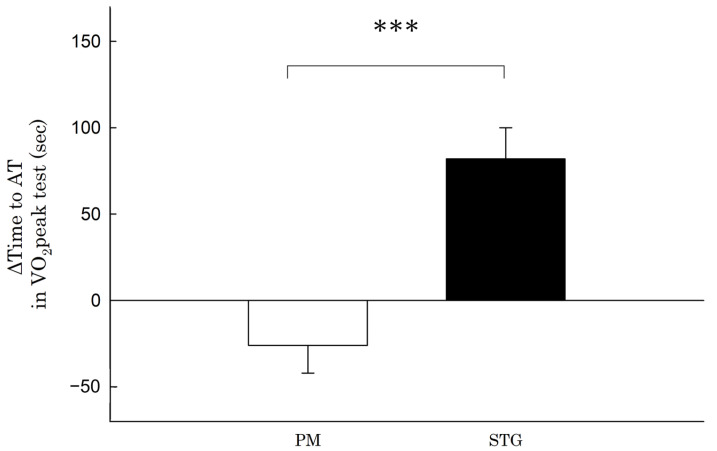
Change in the time to anaerobic threshold in the VO_2_peak test. PM: physical mixture; STGs: structured triglycerides; AT: anaerobic threshold. *** Statistically significant difference from the value in the STG group (*p* < 0.001).

Compared with the difference in TTE in the VO_2_peak test before and after the intervention period in the PM group (−10 ± 63 s), that in the STG group (53 ± 53 s) was significantly longer (63 s, 95% CI 6–120, ES = 1.1, *p* < 0.05) (Figure 2). Compared with the difference in exercise duration to AT during the VO_2_peak test before and after the intervention period in the PM group (−26 ± 52 s), that in the STG group (82 ± 55 s) was significantly longer (109 s, 95% CI 57–161, ES = 2.0, *p* < 0.001) (Figure 3).

## 4. Discussion

The objective of this study was to examine the effects of the intake of STGs (interesterified structured lipids EPA and MCTs) for 8 weeks on cardiorespiratory endurance in sedentary men compared with the intake of a PM of EPA and MCTs. We found that increases in TTE in the VO_2_peak test and AT were greater after consumption of interesterified structured lipids EPA and MCTs than after consumption of the PM of EPA and MCT. However, VO_2_peak during the intervention period and TTE in the endurance test did not differ between the groups.

The consumption of STGs extended the time to reach AT, suggesting the possibility that lipid utilization was enhanced, while carbohydrate utilization was conserved during low-intensity exercise. Indeed, a previous study reported increases in lipid oxidation during low-intensity exercise after the intake of MCTs for 2 weeks in sedentary men and women [25]. In the present study, there was no difference in TTE between before and after the intervention in the PM group (−10 ± 63 s), but TTE was longer after the intervention compared with before in the STG group (by 53 ± 53 s, *p* < 0.05), probably because carbohydrate utilization was conserved during low-intensity exercise. Peroxisome proliferator-activated receptor-γ coactivator (PGC-1a) is a key regulator of mitochondrial biogenesis, and EPA has been shown to stimulate mitochondrial biogenesis [27]. Thus, the present study suggested that an increase in mitochondria via the PGC-1a pathway may be involved in increases in TTE in the VO_2_peak test and AT [28]. Moreover, another possible mechanism could be that EPA and DHA increase the deformability of red blood cells and oxygen supply to muscles [29]. Since EPA and DHA intake increased lipid oxidation [30,31], it is possible that the increased exercise time may have resulted from the conservation of carbohydrate use during exercise.

In this study, VO_2_peak was decreased after the intervention in both groups. One possible reason for this finding was that participants had fewer opportunities to go outside due to the spread of COVID-19. Also, because this study was conducted from August to October 2021, many of the participants (i.e., university students) were on summer vacation. We examined the amount of physical activity before and after the intervention period using the Global Physical Activity Questionnaire [32,33,34,35] and found significant decreases in time spent engaged in high-intensity exercise after supplement intake (before and after the intervention period, 47.9 ± 51.11 min and 21.8 ± 36.0 min, respectively), and significant increases in time spent sitting or reclining (before and after the intervention period, 438.9 ± 190.8 min and 527.4 ± 199.6 min, respectively) in the overall participant population. Increases [13,30] or no changes in VO_2_peak [15,36], but no decreases in VO_2_peak, were previously reported after continuous intake of omega-3 fatty acids. Also, intake of omega-3 fatty acids is reported to be associated with increases in oxygen uptake efficiency, as well as subsequent decreases in oxygen uptake and heart rate during exercise of the same intensity [12,15,37]. However, the findings of previous studies do not suggest that the intake of omega-3 fatty acids was a cause.

In the PM group, the AT was significantly lower after the intervention period than before. Interestingly, in the STG group, there was no difference in AT between before and after the intervention period, despite significant decreases in VO_2_peak in the overall participant population. In addition, the ratio of AT to VO_2_peak (AT (%VO_2_peak)) in the STG group was significantly higher after the intervention period than before (before, 59 ± 9%; after 65 ± 5%; difference, 6 ± 5%; *p* < 0.01), whereas that in the PM group was not significantly different (before, 57 ± 9%; after 57 ± 6%; difference, 0 ± 6%; not significant). Also, the changes in AT (%VO_2_peak) between before and after the intervention period were significantly greater in the STG group than in the PM group (*p* < 0.05). Because greater AT means that one can perform aerobic exercise for a longer time, increases in lipid oxidation during exercise below AT may have contributed to the enhanced endurance observed in the STG group.

The TTE in the endurance test was not increased by the supplement intake in either group in this study. TTE in the endurance test after intake of omega-3 fatty acids was examined previously. Nosaka et al. examined 8 active adult athletes (men and women) who consumed MCTs or LCTs for 2 weeks and found that TTE in exercise performed at 80% of VO_2_peak was significantly longer in the MCT group than in the LCT group [21]. Also, the same research group examined 8 sedentary women who consumed a mixture of MCTs and carbohydrates or carbohydrates only for 2 weeks and found that TTE in exercise performed at 70% of VO_2_peak was significantly longer in the group who consumed the mixture. In contrast, Peoples et al. examined healthy male cyclists and found that TTE in cycle ergometer exercise did not significantly differ between those who consumed omega-3 fatty acids for 8 weeks and those who consumed a placebo [38]. Oostenbrug et al. examined male cyclists and found that exercise performance in 1 h cycle ergometer exercise did not significantly differ between those who consumed omega-3 fatty acids for 3 weeks and those who consumed a placebo [26]. One possible reason that we did not find any change in the endurance test by TTE in this study seems to be too-high exercise intensity. Our results showed that VO_2_peak in both groups decreased before and after the intervention, while AT did not change only in the STG group. We believe this may be due to the increased rate of lipid burning during exercise at 50% of VO_2_peak during the endurance performance test in the STG group. Thus, depending on the intensity of exercise in the endurance performance test (such as below AT levels), there is the possibility to find the effect of supplementation on TTE. Taken together, the effect of both STGs and PM on TTE in endurance tests remains unclear and, thus, needs to be investigated further.

## 5. Conclusions

This study showed that the intake of interesterified structured lipids EPA and MCTs for 8 weeks increased TTE in the VO_2_peak test and AT compared with the intake of the PM of EPA and MCT, suggesting that the intake of interesterified structured lipids EPA and MCTs enhanced lipid utilization during low- and medium-intensity exercise. The limitations of this study are that decreases in VO_2_peak were observed during the intervention in both groups and that there were no differences in TTE in the endurance tests (i.e., endurance performance) between the groups. In addition, since the sample size of this study was relatively small, further studies with larger sample sizes will be necessary.

## Figures and Tables

**Table 1:** Physical characteristics of the participants.

	Overall (*n* = 19) Mean (SEM)	PM (*n* = 10) Mean (SEM)	STG (*n* = 9) Mean (SEM)	*p*
Age (y)	20 (1)	20 (1)	20 (1)	n.s
Height (cm)	171.5 (1.4)	171.3 (2.2)	171.7 (2.2)	n.s
Weight (kg)	67.1 (2.0)	67.3 (3.3)	66.9 (3.3)	n.s
Body mass index (kg/m^2^)	22.8 (0.7)	22.9 (1.0)	22.8 (1.0)	n.s

PM: physical mixture; STG: structured triglycerides; n.s: not significant between PM vs. STG.

**Table 2:** Changes in the VO_2_peak test and endurance test outcomes from baseline to week 8.

	STG (*n* = 9)	PM (*n* = 10)	Between-Group Difference		
	Mean (SEM)	Mean (SEM)	Mean (95% CI)	ES	*p*
VO_2_peak (mL/kg/min)					
Before	42.4 (1.6)	44.3 (2.3)			
After	39.6 (1.0)	39.5 (2.4)			
Within-group difference	−2.8 (1.1)	−4.8 (1.1)	2.0 (−1.2 to 5.3)	0.61	0.20
AT (mL/kg/min)					
Before	25.0 (1.5)	25.4 (2.2)			
After	25.7 (1.0)	22.5 (1.7)			
Within-group difference	0.6 (0.8)	−2.9 (0.8)	3.5 (1.1 to 6.0)	1.42	<0.01
TTE in VO_2_peak test (s)					
Before	839 (26)	849 (38)			
After	892 (29)	839 (44)			
Within-group difference	53 (18)	−10 (20)	63 (6 to 120)	1.1	<0.05
Time to AT in VO_2_peak test (s)					
Before	372 (27)	371 (33)			
After	455 (30)	344 (37)			
Within-group difference	82 (18)	−26 (16)	109 (57 to 161)	2.0	<0.001
TTE in endurance performance test at a workload to 80% VO_2_peak (s)					
Before	726 (171)	648 (106)			
After	883 (162)	864 (97)			
Within-group difference	157 (160)	216 (73)	−58 (−417 to 300)	−0.16	0.74
Total workload in endurance performance test (J/kg)					
Before	5383 (433)	5397 (244)			
After	6140 (508)	5716 (365)			
Within-group difference	757 (454)	319 (234)	439 (−605 to 1483)	0.42	0.41
Total workload at 50%VO_2_peak in endurance performance test (J/kg)					
Before	3512 (154)	3714 (223)			
After	3683 (111)	3514 (282)			
Within-group difference	170 (100)	−200 (109)	370 (56 to 684)	1.15	<0.05
Total workload at 80%VO_2_peak in endurance performance test (J/kg)					
Before	1870 (428)	1684 (208)			
After	2457 (479)	2202 (242)			
Within-group difference	587 (443)	518 (189)	69 (−911 to 1048)	0.07	0.890

AT: anaerobic threshold; VO_2_peak: peak oxygen uptake; TTE: time to exhaustion; SEM: standard error of the mean; CI: confidence interval; ES: effect size.

## Data Availability

The datasets used and analyzed during the current study are available from the corresponding author upon reasonable request.

## Abbreviations

AT: anaerobic threshold; BMI: body mass index; DHA: dDocosahexaenoic acid; EPA: eicosapentaenoic acid; FAO: facilitate fatty acid beta-oxidation; FFQg: Food Frequency Questionnaire Based on Food Groups; LCTs: long-chain triglycerides; MCTs: medium-chain triglycerides; PM: physical mixture; STGs: structured triglycerides; TTE: time to exhaustion; VO_2_peak: peak oxygen uptake.
